# Lycopene, Tomato Products, and Prostate Cancer Incidence: A Review and Reassessment in the PSA Screening Era

**DOI:** 10.1155/2012/271063

**Published:** 2012-05-28

**Authors:** Melissa Y. Wei, Edward L. Giovannucci

**Affiliations:** ^1^Department of Medicine, Emory University School of Medicine, Atlanta, GA 30303, USA; ^2^Department of Nutrition and Department of Epidemiology, Harvard School of Public Health, Boston, MA, USA; ^3^Channing Laboratory, Department of Medicine, Brigham and Women's Hospital and Harvard Medical School, Boston, MA 02115, USA

## Abstract

Lycopene has been proposed to protect against prostate cancer through various properties including decreased lipid oxidation, inhibition of cancer cell proliferation, and most notably potent antioxidant properties. Epidemiologic studies on the association between lycopene and prostate cancer incidence have yielded mixed results. Detection of an association has been complicated by unique epidemiologic considerations including the measurement of lycopene and its major source in the diet, tomato products, and assessment of prostate cancer incidence and progression. Understanding this association has been further challenging in the prostate-specific antigen (PSA) screening era. PSA screening has increased the detection of prostate cancer, including a variety of relatively indolent cancers. This paper examines the lycopene and prostate cancer association in light of epidemiologic methodologic issues with particular emphasis on the effect of PSA screening on this association.

## 1. Introduction

Several chemoprotective properties of lycopene on prostate cancer have been proposed, including potent antioxidant properties, decreased lipid oxidation, inhibition of cancerous cell proliferation at the G0-G1 cell cycle transition, and protection of lipoproteins and DNA [1, 2]. These mechanistic studies have stimulated the examination of lycopene and its primary source, tomato products, on risk of prostate cancer. However, studies on lycopene and tomato intake and prostate cancer incidence have yielded mixed results. The study of this relationship has been complicated by unique and challenging epidemiologic considerations in the measurement of lycopene and on the influence of prostate-specific antigen (PSA) screening on prostate cancer incidence and progression. As with all epidemiologic studies, validity depends on the quality of the methodology. This paper briefly describes the methodology essential for conducting studies on the association between lycopene and prostate cancer incidence and provides an updated review of studies of lycopene and tomato products with prostate cancer risk.

## 2. Measurement of Lycopene and Tomato Products

Lycopene is a carotenoid devoid of vitamin A activity. The major source by far, particularly in Western populations, is tomato and tomato products; a few other foods such as watermelons and pink grapefruit also contain lycopene. In epidemiologic studies, approaches to assess an individual's lycopene intake or status include studies that estimate intake of lycopene (based on reported intake of foods and food content of lycopene from food composition databases), studies that assess tomato product intake as a surrogate of lycopene intake, and studies that measure lycopene levels in the serum or plasma. An issue that is not unique for lycopene, but perhaps of special importance for this carotenoid, is the variable absorption of lycopene from different food sources. In particular, cooking in an oil medium substantially enhances bioavailability of lycopene in the intestine because lycopene is highly bound to plant source matrices and is highly lipophilic. The measure of lycopene in the serum has theoretical advantages of accounting for absorption and not relying on study participants' food recall and accuracy of food composition tables. On the other hand, serum studies have frequently relied on a single measure, and how well a single measurement reflects long-term intake is not entirely clear. Also, since lycopene comes largely from tomato sources, circulating lycopene level may be acting as a surrogate of tomato product intake, and other components of tomatoes may account for any observed association with cancer risk. Finally, a population that consumes overall low levels of lycopene or similar levels of lycopene across individuals may result in insufficient contrast between high and low consumers.

## 3. The Influence of PSA Screening on Clinical and Epidemiologic Aspects of Prostate Cancer Incidence

PSA release into the serum occurs with tissue breakdown between the prostate gland lumen and capillaries. The original purpose of the PSA measurement was to monitor prostate cancer progression and recurrence. The Food and Drug Administration approved PSA testing for monitoring disease status in men with prostate cancer in 1987 and expanded its use to diagnosing prostate cancer in 1992. This approval was followed by professional society guidelines that supported the use of PSA testing for prostate cancer screening. Consequently, PSA became widely used as a screening test in the United States and increasingly in other countries. As a screening modality, the sensitivity and specificity of PSA varies based on the cut-off. For example, a PSA level of 3 ng/mL has a sensitivity of 32% for detecting any prostate cancer and 68% for high-grade prostate cancer and a specificity of 85%. If this level were increased to 4 ng/mL the sensitivity would decrease to 21% for any prostate cancer and 51% for high grade prostate cancer but specificity would improve to 91% [3].

Elevated serum PSA may precede invasive carcinoma by a minimum of 5–10 years. Thus, PSA testing enabled earlier detection of prostate cancer. The rate of first-time PSA testing was strongly correlated with prostate cancer incidence rates. With the onset of PSA for screening, prostate cancer incidence increased and peaked in 1992 and declined thereafter [4]. Localized, nonmetastatic cancers accounted for most of the increased incidence. Prior to widespread PSA use for screening, prostate cancer diagnosis was largely prompted by physical exam findings of an enlarged prostate or symptoms ranging from urinary incontinence to more advanced spinal cord compression and bony pain from metastasis; therefore, prostate cancer was mostly detected in relatively advanced stages. Invasive carcinoma prevalence increases with age: 2% for men in their 30 s compared with 64% for men in their 70s [5]. One-third of men under age 80 will have prostate cancer detected on autopsy. The lifetime risk of prostate cancer is 16% while the risk of mortality from prostate cancer is 2.9% [6]. Thus a “PSA screening era” beginning in 1988, FDA approved in 1992, and peaking between 1992 and 1998 has been referred to as a period in which prostate cancers diagnosed by serum PSA alone encompassed a variety of stages of prostate cancers, including clinically indolent cancers [4, 7].

PSA testing revolutionized the detection of prostate cancer but was not without unexpected consequences. In addition to diagnosing biologically indolent cancers, PSA elevation occurs with benign conditions including benign prostatic hypertrophy, prostatitis, subclinical inflammation, ejaculation, digital rectal exams (potentially performed just prior to patients having their PSA lab drawn), perineal trauma, prostatic infarction, urinary retention, biopsy, and transurethral resection of the prostate. The number of false positives is high, leading to numerous negative biopsies.

The influence of PSA on prostate cancer mortality has been controversial, with randomized trials not yielding a clear answer. However, undoubtedly PSA screening has caused an increase in the number of indolent cancers being treated aggressively and ultimately led to increased morbidity from side effects of treatment. The majority of newly diagnosed prostate cancers were clinically localized and unlikely clinically significant to involve aggressive medical and surgical therapy such as radical prostatectomy with radiation ablation intended to cure early-stage cancers. For these reasons, in 2011 the United States Preventive Services Task Force recommended against PSA screening for prostate cancer regardless of age, race/ethnicity, and family history.

Beyond the clinical consequences, PSA screening has altered the landscape of prostate cancer epidemiology. Many more cancers are diagnosed, including a substantial proportion of relatively indolent cancers, and the cancers are diagnosed earlier in their natural history, often before evidence of aggressive behavior is manifested. Thus, depending on at what stage and on what subtype of prostate cancer a risk factor may be acting, the relationship between this risk factor and prostate cancer risk may differ in populations exposed or not exposed to widespread PSA screening [8]. By increasing the heterogeneity in prostate cancers being diagnosed, PSA screening has added complexity to the epidemiologic study of prostate cancer.

## 4. Epidemiologic Studies

### 4.1. Clinical Trials

A number of randomized clinical trials have examined lycopene and prostate cancer progression and mortality in men diagnosed with prostate cancer [9]. The randomized studies have been small and inconclusive [10]. In a double-blind randomized placebo-controlled trial, 105 African American male veterans recommended for biopsy to detect prostate cancer were administered tomato sauce containing 30 mg/day of lycopene or placebo over 21 days [11]. PSA and lycopene levels were measured, and the group randomized to lycopene had an increase in serum lycopene and decrease in PSA while the placebo group had the reverse, with a decrease in serum lycopene and increase in PSA. This study did not report a significant decrease in prostate cancer risk for individuals administered lycopene, but the study duration of 21 days was likely inadequate to significantly influence prostate cancer risk.

Two other studies examined PSA levels in relation to lycopene administration [12]. One study reported a decline in PSA in the lycopene as well as placebo group after 1 month of intervention but return to baseline PSA levels for both groups after 4 months of followup [10]. Schwartz et al. [12] did not report a decrease in PSA levels among individuals administered lycopene. In general, the clinical trials have been considerably limited by size, length of study duration, and other methodological issues and do not provide strong support or refutation of an association between lycopene and prostate cancer risk. No adequately sized randomized studies of lycopene for prostate cancer prevention have been conducted.

### 4.2. Prospective Dietary Studies

Prospective and nested case control studies have been published previously in qualitative [13, 14] and quantitative reviews [15]. In a meta-analysis of prospective studies up to 2003 [15], high intake of raw but not cooked tomatoes was associated with a decreased risk of prostate cancer (relative risk (RR) 0.71, 95% confidence interval (CI): 0.57–0.87). Subsequent cohort studies on dietary lycopene intake [16, 17] have not reported significant inverse associations with prostate cancer risk. However, these studies were conducted in the post-PSA era that likely encompassed a heterogeneous group of prostate cancers that included latent and incident cancers.

Among prospective dietary studies, four [18–21] of six cohorts report an inverse relationship between lycopene or tomato consumption and prostate cancer incidence. The largest and only study with multiple assessments of diet was conducted in the Health Professionals Follow-up Study (HPFS) [18, 19]. The HPFS first reported an inverse association between lycopene intake in 1986 with prostate cancer diagnosed between 1986 and 1992: RR for high versus low quintile of intake = 0.79 (95% CI: 0.64–0.99, *P*_trend_ = 0.04) [18]. High intake of tomato-based products was associated with a 35% decreased risk of total prostate cancer (RR 0.65, 95% CI: 0.44–0.95) and 53% decreased risk of advanced stage prostate cancer (RR 0.47, 95% CI: 0.22–1.00; *P*_trend_ = 0.03).

The HPFS analysis was updated for prostate cancer cases between 1992 and 1998 using cumulative average updated intakes (i.e., averaging intake from all the dietary questionnaires up to the time period of risk) of lycopene from 1986 to 1998 with a similar inverse association detected: RR = 0.83 (95% CI: 0.70–0.98, *P*_trend_ = 0.02) [19]. The HPFS assessed dietary intake every four years, and the timing of intake in relation to period of risk for prostate cancer was assessed. When baseline lycopene intake in 1986 was evaluated for prostate cancer cases during the entire follow-up period, no significant association was seen. However, statistically significant inverse associations were found when using the questionnaire closest in time to the time period of risk (RR for high versus low lycopene intake = 0.84, 95% CI: 0.74–0.96, *P*_trend_ = 0.02) and cumulative average updated lycopene intake (RR for high versus low lycopene intake = 0.84, 95% CI: 0.73–0.96, *P*_trend_ = 0.003). These findings suggest that lycopene may be acting relatively late in the carcinogenic process. Alternatively, a single measurement of dietary intake at baseline may not be the best measurement to reflect the potential impact of lycopene in altering prostate carcinogenesis, compared with multiple updated dietary measurements. Individual tomato products were examined in relation to prostate cancer risk, and the strength of the association corresponded to association of the food item with serum lycopene levels, which were concurrently available in the HFPS. For example, tomato sauce, the most bioavailable form of lycopene, was most strongly related to decreased prostate cancer risk (RR for ≥2 servings/week versus <1 serving/month = 0.77, 95% CI: 0.66–0.90), followed by tomato and pizza but not tomato juice. There was an even stronger association for advanced prostate cancer: RR for ≥2 servings/week versus <1 serving/month of tomato sauce = 0.65 (95% CI: 0.42–0.99, *P*_trend_ = 0.02).

A similar magnitude decrease in prostate cancer was reported in the California Seventh Day Adventist cohort between 1974 and 1982 for men with high tomato consumption [21]. High intake of tomatoes was associated with a statistically significant 40% decreased risk of prostate cancer (RR 0.60, 95% CI: 0.37–0.97). In another study, a 50% lower risk of prostate cancer was reported for high compared with low tomato consumption in US men between 1987 and 1990: RR 0.50 (95% CI: 0.30–0.90, *P*_trend_ = 0.03), but few details were provided [20].

Dietary cohort studies that did not report associations with prostate cancer incidence frequently had lower lycopene and tomato intake compared with studies that report an association. A dietary cohort study based in The Netherlands between 1986 to 1992 did not report an association between tomato intake and prostate cancer incidence [22]. While this cohort took place between the same time period as the initial HPFS analysis, tomato consumption was low compared with the HPFS. In addition, consumption of tomato-based products, which contain more bioavailable lycopene, was not specifically addressed.

A diet-based cohort study of individuals in the Prostate, Lung, Colorectal, and Ovarian Screening Trial [17] examined intakes of lycopene and top food sources of lycopene but did not find inverse associations for lycopene, raw tomatoes, canned tomatoes, or other processed tomato products (ketchup, tomato sauce, pizza, lasagna, tomato and vegetable juice, chili) with total or nonadvanced prostate cases. Predominately white men (90.7%) enrolled in this study between 1993 and 2001 received baseline PSA screening or digital rectal examination and completed annual questionnaires. Men with a PSA level >4 ng/mL or digital rectal examination concerning for prostate cancer were referred to their medical provider for further diagnostic evaluation and staging of prostate cancer. The majority (92%) of prostate cancers were confirmed for stage and grade. Nonadvanced disease (Gleason score <7 or stage I or II) comprised 61% of total prostate cancer cases and was not associated with total lycopene intake or lycopene from processed foods. Greater consumption of spaghetti/tomato sauce and pizza was associated with decreased incidence of advanced disease but did not reach statistical significance (RR 0.81, 95% CI: 0.57–1.16 and RR 0.79, 95% CI: 0.56–1.10, respectively). The association of tomato products with advanced but not nonadvanced prostate cancer suggests a possible stronger role for lycopene in advanced disease. Limitations of this study include the assessment of lycopene intake from a single baseline measurement. In addition, intake for all but raw tomatoes was low in this population, with no more than two servings per week (mean total lycopene intake 11,511 and standard deviation 8,498 *μ*g/d). Further, a large portion of prostate cancer cases were diagnosed by initial PSA screening, and total prostate cancers reflected a heterogeneous mix of mostly nonadvanced prostate cancer cases.

Dietary lycopene was not reported to have an association with prostate cancer risk in a prospective study from the Prostate Cancer Prevention Trial [16]. The PCPT originated as a randomized trial of finasteride but was converted to a prospective observational study. Diet was assessed one year after randomization in 1994. Men underwent annual screening for prostate cancer using digital rectal examination and PSA. Men with an abnormal digital rectal examination or PSA level 4 ng/mL or greater were encouraged to receive a prostate biopsy. At the final study year all men not previously diagnosed with prostate cancer were offered prostate biopsies. This resulted in the inclusion of incidental cases of prostate cancer that contributed to the substantial 24.8% of prostate cancer cases diagnosed in men originally randomized to the control group. The majority of cancers were localized and detected by screening or incidentally discovered, and it is possible that low-grade cancers have different characteristics from high-grade cancers. Prior studies of lycopene intake have suggested stronger inverse associations with advanced prostate cancer [18]. In addition, it may be necessary to assess lycopene through simple updated or cumulative average updated intakes rather than a single baseline measurement.

### 4.3. Prospective Serum and Plasma Studies

 A number of studies have examined serum or plasma lycopene from biobanks in relation to subsequent prostate cancer risk. The studies were typically nested case control in design; incident cases and matched cancer-free controls over the follow-up period were identified, and serum or plasma lycopene was measured in the banked biospecimens. In a meta-analysis of prospective studies up to 2003 [15], high serum lycopene levels were associated with significant decreased risk of prostate cancer: RR = 0.78 (95% CI: 0.61–1.00). Subsequent studies on serum lycopene levels conducted in the post-PSA era [23–28] have not reported significant inverse associations with total prostate cancer risk.

One of the earliest studies to report an inverse association between serum lycopene and prostate cancer risk was a nested case control study of 103 prostate cancer cases matched with 103 controls among 25,802 male residents of Washington County, MD who donated blood in 1974 [29]. A nonsignificant 50% reduction in prostate cancer risk was reported (OR 0.50; 95% CI: 0.20–1.29). Two of the largest nested case control studies reported inverse associations between plasma lycopene and prostate cancer risk [30, 31]. Plasma lycopene levels were higher in these multicentered US cohorts compared with other studies, which may reflect the higher education level in these populations. A nested case control study within the Physicians' Health Study, a randomized, placebo-controlled trial of aspirin and *β*-carotene, assessed incident prostate cancer cases in 578 men compared with 1294 age- and smoking-status-matched controls [30]. Plasma lycopene was lower in cases than controls. Men with higher plasma lycopene had a borderline significant decreased risk of prostate cancer (highest quintile RR = 0.75; 95% CI: 0.54–1.06; *P*_trend_ = 0.05). There was a significantly greater decreased risk for aggressive (high stage or high grade) prostate cancer: highest quintile plasma lycopene, RR = 0.56 (95% CI: 0.34–0.91; *P*_trend_ = 0.05). These associations were not confounded by covariates including age, smoking status, body mass index, physical activity, alcohol intake, multivitamin use, or plasma total cholesterol level.

In a nested case control study within the prospective HPFS cohort, 450 incident cases of prostate cancer diagnosed between 1993 and 1998 were matched with 450 controls by age, time, month, season, and year of blood donation [31]. A nonsignificant inverse association was reported for plasma lycopene and risk of prostate cancer: RR for highest versus lowest quintile, 0.66 (95% CI: 0.38–1.13). This association was statistically significant for men older than 65 years at time of plasma donation: RR for highest versus lowest quintile, 0.47 (95% CI: 0.23–0.98), but was not observed for younger men.

Serum lycopene was recently assessed in a nested case control study within the Prostate Cancer Prevention Trial (PCPT) [28]. There was no association between serum lycopene and prostate cancer incidence, but incidentally diagnosed prostate cancer cases by end-of-study biopsies were analyzed alongside prostate cases diagnosed by screening. In a reanalysis that included only cancers diagnosed from abnormal screening there was a significant inverse association between high serum lycopene and prostate cancer [7]. The cancers assessed by end-of-study biopsy were relatively static (e.g., no PSA elevation or sign of clinical progression) during the study period, so may not be appropriately considered as “incident” prostate cancer.

Several other serum lycopene studies reported nonsignificant inverse associations [23–25, 27] or no association [26, 32] with prostate cancer risk. However, these studies were conducted in the post-PSA era that likely encompassed a heterogeneous group of prostate cancers that included latent and incident cancers. As with the PCPT, an association with lycopene could be missed. In the large European study (EPIC) by Key et al., a statistically significant inverse association was observed for cases diagnosed at an advanced stage [26]. In this study, men in the highest versus lowest quintile of lycopene level had a RR of 0.40 (95% CI: 0.19–0.88).

### 4.4. Case Control Dietary Studies

Several studies retrospectively examined the association between tomatoes, tomato-based products or lycopene, and prostate cancer risk, with mixed results. In a meta-analysis of case control studies through 2003 [15], high intakes of raw tomatoes, cooked tomatoes, and lycopene were not associated with decreased prostate cancer risk.

A strong inverse dose-response relationship between lycopene intake and histopathologically confirmed prostate adenocarcinoma risk was reported in a case control study of Chinese men [33]. Lycopene intake was assessed using a reproducible and validated 130-item food frequency questionnaire for elderly men in China [34]. For lycopene intakes of 1609–3081, 3081–4917, and >4917 *μ*g/d, the RRs of prostate cancer compared with lycopene intake <1609 *μ*g/d were 0.47 (95% CI: 0.25–0.86), 0.40 (95% CI: 0.21–0.77), and 0.17 (95% CI: 0.08–0.39), respectively. The RRs reported for lycopene and prostate cancer in this study were stronger than some prior studies. This study also examined green tea and vegetable and fruit intake and reported strong, significant inverse associations for all these associations. The incidence of prostate cancer is lower in developing countries such as China, compared with Western countries. In China PSA screening is not as commonly widely used compared with Western countries. Prostate cancer is thus usually diagnosed at more advanced stages. While further information and stratification based on prostate cancer grade and staging were not provided in this study, the lower prevalence of PSA screening practices in China compared with the USA suggests prostate cancer cases in this cohort were more likely to be at advanced stages. The strong association between lycopene and probable advanced prostate cancer suggests a role for lycopene in influencing risk of aggressive cancer.

A significant inverse association between tomato intake and prostate cancer was similarly reported in a case control study of 617 Canadian men with prostate cancer and 636 age-matched controls conducted between 1989 and 1993. The RR of prostate cancer was 0.64 (95% CI: 0.45–0.91) for tomato intake >73 g/day compared with <24 g/day. There was no significant association reported for lycopene intake and prostate cancer [35].

In a case control study of 130 prostate cancer cases in Iranian men, tomato consumption of greater than 100 g/week was nonsignificantly inversely associated with decreased prostate cancer risk (RR 0.45; 95% CI: 0.09–2.12) [36]. Food intake was assessed based on the past two months of intake, and tomato intake questions included tomato extract and dressing. However, it is unclear whether this tomato group included both raw and processed tomatoes.

An additional study reported a nonsignificant inverse association between dietary lycopene and prostate cancer risk [37] while several reported no association [38–40].

### 4.5. Case Control Serum and Plasma Studies

 Three case control studies of plasma lycopene reported strong inverse associations with histopathologically confirmed prostate cancer [41–43]. One study of non-Hispanic Caucasian men used high-pressure liquid chromatography (HPLC) to examine plasma lycopene isoforms [42]. An inverse association was reported for *cis*-lycopene-1 only. *cis*-lycopenes 2 through 5 individually and in sum as total *cis*-lycopene and *trans*-lycopene were not associated with prostate cancer risk. This study suggests the structural type of lycopene measured may influence the ability to detect an association, as one cis isomer but not total *cis*- or *trans*-lycopene was associated with decreased prostate cancer risk. Data on individual structural lycopene isomers have been limited. However, a study in the HPFS found that the isomers were highly correlated with each other, making any specific effects difficult to distinguish [44].

The multicentered case control Third National Health and Nutrition Examination Survey (NHANES III) of U.S. Caucasian and African American men aged 40–79 years reported a significant inverse association between serum lycopene and aggressive prostate cancer (highest compared with lowest quartile RR = 0.37; 95% CI: 0.15–0.94; *P*_trend_ = 0.04) and nonsignificant association between serum lycopene and prostate cancer (highest compared with lowest quartile RR = 0.65; 95% CI: 0.36–1.15; *P*_trend_ = 0.09) [45]. Caution should be used in interpreting results from case control plasma or serum studies because the cancers could possibly be influencing lycopene level, resulting in reverse causation.

## 5. Lycopene and Prostate Cancer Associations: Conclusions

This paper focused on prostate cancer incidence that may or may not have been detected by initial PSA screening and highlights differences in the association with lycopene based on prostate cancers initially screened with PSA testing compared with cancers diagnosed in more advanced stages. Elevated PSA may be attributed to a number of benign factors, including the highly prevalent benign prostatic hypertrophy in older men, and should not be used in isolation along with single serum measurements for the diagnosis of prostate cancer. Randomized interventions of lycopene and prostate cancer risk have been limited in scope, and some used PSA as an endpoint [10, 11]. Thus, trials do not provide strong support either for or against a causal association.

The epidemiologic literature on lycopene intake or level and prostate cancer based on observational studies has been inconsistent overall. Earlier studies had appeared more promising but some recent studies are not supportive. There are several potential explanations for this pattern. One potential explanation is that there was a relative over-reporting and publishing of positive studies in the earlier years, followed by a correction of this publication bias as the hypothesis grew in interest and null studies were published. If so, then it may be concluded that there is unlikely to be a causal connection between lycopene intake and risk of prostate cancer.

An alternative possibility is that the earlier studies were conducted largely before the onset of PSA screening, where diagnosis of prostate cancer usually implied a period of increasing aggressive behavior leading to the diagnosis. Thus, the exposure was linked to the development of aggressive behavior in cancers with biologic potential to progress. In the PSA era, 1992–1998 with peak in 1992 following FDA approval as a screening test for prostate cancer, the diagnosis of prostate cancer is not typically linked to aggressive behavior [4, 7]. An example of this phenomenon may be the subgroup of cancers in the PCPT that were diagnosed at end of study biopsy. There was a high prevalence of undiagnosed prostate cancer, even among the youngest group of men in the study (55–59 years), which suggests that most of the cancers eventually diagnosed during the study period were present at baseline. Throughout the 7-year followup, the cancers diagnosed at end-of-study biopsy showed no evidence of clinical or biochemical progression [28]. Prior studies have shown that most cancers, even many with high-grade Gleason scores, do not progress over prolonged time, and it is well established that only a fraction of prostate cancers result in the most advanced and clinically significant stage and mortality [46]. Thus, the majority of these cancers was likely present at the onset of the study and may be considered static cancers. In fact, when reanalyzed as a case-only study, higher serum lycopene levels in the PCPT appeared to be inversely associated preferentially with cancers that showed evidence of progression relative to cancers that showed no indication of progression. In modeling two patterns of prostate cancer progression, one with low lycopene exposure and rapid tumor growth that reaches a threshold PSA level for clinical diagnosis and the second with high lycopene exposure and slow progression, the latter may be diagnosed at a much later time through an incidental random biopsy rather than PSA. Thus, asymptomatic cancers diagnosed at the end-of-study by biopsy may signify cancers that were inhibited rather than incident cancer. Thus, a possible interpretation is that high levels of lycopene may have inhibited some existing cancers to undergo progression.

Some evidence supports the premise that PSA screening and type of tumor endpoint are critical. For example, the HPFS was analyzed before and after peak PSA testing in the early 1990s. Before PSA testing (1986–1992) tomato sauce intake was inversely associated with prostate cancer incidence and stronger for advanced stage cancers. While the association for total prostate cancer incidence was attenuated during the PSA era (1992–1998), the association with metastatic prostate cancer persisted. In addition, in the large EPIC study [26] serum lycopene level was inversely associated with risk of advanced stage prostate cancer but not nonadvanced prostate cancer.

Additional factors may contribute to the heterogeneity in the literature. As discussed above, studies based on intake are limited by the assessment of intake, food composition databases, and differences in bioavailability. Future studies may be improved by better taking into account bioavailability differences among diverse foods. Prospective studies are preferable to avoid various biases, such as recall bias, or reverse causation in studies of circulating lycopene. With increasing use of PSA, it is becoming increasingly difficult to examine advanced stage prostate cancer, at least in some populations. Examining potential mediators or markers of aggressive behavior in tumor tissue may be another useful approach in the further study of lycopene and prostate cancer risk.

## Abbreviations

CI: Confidence interval
HPFS: Health Professionals Follow-up Study
OR: Odds ratio
PCPT: Prostate Cancer Prevention Trial
PSA: Prostate-specific antigen
RR: Relative risk.
